# Health-related quality of life of trans people using a specialized health service in Manaus, Amazonas state, Brazil, 2023: a cross-sectional study

**DOI:** 10.1590/S2237-96222024v33e2024361.especial.en

**Published:** 2025-01-10

**Authors:** Erivan Clementino Gualberto, Açucena Amâncio Dall’Alba, André Luiz Machado das Neves, David Câmara Gurgel do Amaral, Aline Barreto Soares, Fernando José Herkrath, Ana Paula Corrêa de Queiroz Herkrath

**Affiliations:** 1 Universidade Federal do Amazonas, Faculdade de Odontologia, Manaus, AM, Brazil; 2 Universidade do Estado do Amazonas, Escola Superior de Ciências da Saúde, Manaus, AM, Brazil; 3 Fundação Oswaldo Cruz, Instituto Leônidas e Maria Deane, Manaus, AM, Brazil

**Keywords:** Personas Transgénero, Calidad de Vida Relacionada con la Salud, Servicios de Salud para Personas Transgénero, Estudios Transversales, Trans People, Health-Related Quality of Life, Health Services for Transgender People, Cross-Sectional Studies

## Abstract

**Objective:**

To assess health-related quality of life and associated factors among trans people.

**Methods:**

This was a cross-sectional study conducted in 2023. The 12-Item Short- Form Health Survey was used with trans women, trans men, *travestis*, and non-binary people who were users of a health service in Manaus, capital city of Amazonas state.

**Results:**

A total of 71 participants were included: 36 trans women; 29 trans men; 2 *travestis*; and 4 non-binary people, with a mean age 30.1 years. The physical component scored 44.6±7.8, and the mental component scored 38.4±11.1. The emotional aspect with a score of 16.0±1.1, was the most affected domain. Paid employment was associated with better mental quality of life, while having lived in street situations was associated with worse physical quality of life, with mean differences of 4.4 (95%CI 2.3;6.9) and -3.3 (95%CI -7.7;2.3) respectively.

**Conclusion:**

Among trans people, the mental component was more affected than the physical, with the greatest impairment observed in the emotional aspect. Socioeconomic characteristics were associated with quality of life.

## Study contributions

**Main results:**

The mental component of the quality of life among the trans people assessed was worse than the physical component, with the greatest impairment in the emotional aspect. Trans people with paid employment reported better quality of life, while having lived in street situations was associated with poorer quality of life.

**Implications for services:**

Quality of life helps to understand self-perceived health status and should be considered as a health outcome. It can highlight unmet needs in health services and promote comprehensive, inclusive and welcoming health care.

**Perspectives:**

Health services and the evaluation of therapeutic interventions for trans people should incorporate self-reported health outcomes. These outcomes enable the assessment of care quality and provide support for health actions that are sensitive to the specific needs of this population.

## RESUMEN

**Objetivo:**

Evaluar la calidad de vida relacionada con la salud y los factores asociados en personas trans.

**Métodos:**

Estudio transversal realizado en 2023, utilizando el instrumento 12-Item Short-Form Health Survey en mujeres trans, hombres trans, travestis y personas no binarias que asisten a un servicio de salud especializado en Manaos.

**Resultados:**

Se incluyeron 71 participantes: 36 mujeres trans; 29 hombres trans; 2 travestis; y 4 personas no binarias, con una edad media de 30,1 años. El componente físico presentó una puntuación de 44,6±7,8, y el componente mental de 38,4±11,1. El dominio emocional, con una puntuación de 16,0±1,1, fue el más afectado. El empleo remunerado se asoció con una mejor calidad de vida mental, mientras que haber vivido en situación de calle se asoció con una peor calidad de vida física, con diferencias medias de 4,4 (IC_95_% 2,3;6,9) y -3,3 (IC_95_% -7,7;-2,3), respectivamente.

**Conclusión:**

En personas trans, el componente mental fue más afectado que el físico, con mayor perjuicio en el dominio emocional. Las características socioeconómicas se asociaron con la calidad de vida.

## INTRODUCTION

Conceptually, the term “trans” encompasses all people who do not identify with traditional dichotomous social expressions of gender.^1^ Given the diversity of experiences among *travestis*, transsexual, and transgender people, “trans” is used as an umbrella term that integrates different identities and functions, representing a diverse group of people who will be referred to in this study as “trans people”.^1^

The global population that identifies as trans is 4.6 in 100,000 people, with a higher percentage for trans women (6.8 per 100,000) compared to trans men (2.6 per 100,000).^2^ In Brazil, gender diversity represents approximately 2% of the adult population. It is estimated that 3 million Brazilians identify as trans. They are, on average, younger (32.9±13.5) when compared to cisgender people (42.2±15.9). The average age of these people may be associated with the lower life expectancy of this population.^3^

Disrespect for the social name and the inability to address specific issues faced by the trans population,^1^ as well as the difficulty of entering the labor market, income, social and family conflicts,^4^-6 permeate the reality of accessing goods and services for trans people. Many health problems within this population are directly and indirectly related to exposure to prejudice, discrimination and violence.^7^ Depression, anxiety, stress, substance abuse that are harmful to health, self-harm and suicide attempts^7^ are common among these individuals. Discriminatory attitudes affect the relationship between doctor and patient and hinder health education and prevention actions.^1^ These exposures negatively impact the quality of life of this population.

The World Health Organization defines quality of life as an individual’s perception of their position in life within the context of their culture and value systems, considering their life goals, expectations, and concerns.^8^ Maintaining the subjectivity and multidimensionality of quality of life, the concept of health-related quality of life emerged, which is defined by the aspects of quality of life that are directly or indirectly related to health.^9^ The understanding of quality of life is focused on the individual’s subjective evaluation^9^ and can be assessed through instruments in the form of questionnaires that investigate the impact of health conditions on the physical, psychological, emotional and social aspects of each person.^10-12^ Throughout this article, the term “quality of life” has been used to refer to the outcome “health-related quality of life”.

Previous studies have shown that the quality of life of trans individuals is worse than that of cisgender individuals.^4,13-15^ Transgender people, when compared to the cisgender population, experience various forms of discrimination that negatively affect their daily lives.^16^ Psychosocial stress has the potential to impact physical, psychological, and well-being domains, further compromising quality of life.^7,16^ Factors such as gender-affirming hormone therapy,^17,18^ a stable romantic relationship,^18^ working or studying^14,18^ are associated with better quality of life scores. Demographic and socioeconomic factors have also been associated with the quality of life of transgender people,^14,17-18^ similar to what is observed in the general population.^19-20^

Studies investigating quality of life in the trans population are still scarce. This study aimed to evaluate health-related quality of life and its associated factors among trans people in Manaus, Amazonas state.

## METHODS

### Study design

This was a cross-sectional study approved by the Research Ethics Committee of the Universidade Federal do Amazonas under opinion 6,315,703, with certificate of submission for ethical appraisal No. 65732222.0.0000.5020. All participants signed the Free and Informed Consent Form, in accordance with the ethical guidelines established by Resolution No. 466/12 of the National Health Council (*Conselho Nacional de Saúde*).

### Setting

The study was conducted from April to December 2023 at the Sexual and Gender Diversity Outpatient Clinic, located at the Codajás Polyclinic, in Manaus, Amazonas state, a reference service of the State Health Department that offers multidisciplinary care to individuals undergoing the transsexualizing process.

### Participants

A convenience sample consisted of individuals aged 18 years and older who identified as trans men, trans women, *travestis*, and non-binary people and were users of the Sexual and Gender Diversity Outpatient Clinic. The exclusion criterion was being under the influence of psychoactive substances or alcohol at the time of the interview, which would prevent the administration of the questionnaire. Participation in the transsexualizing process, the decision to participate, or the therapeutic stage of the process were not considered for eligibility in the study.

People were invited to participate in the study at the reception, while waiting their healthcare appointment. This initial contact was supported by the outpatient clinic staff. Those who agreed to participate and met the eligibility criteria were directed to a private room where informed consent was obtained, and data collection took place.

### Variables, data source and measurement

Data were collected using questionnaires administered through interviews conducted by three trained interviewers. The outcome of the study was health-related quality of life, measured by means of the Short- Form Health Survey (SF-12) in its translated and validated version for Brazilian Portuguese.^21^ The exposures evaluated were demographic and socioeconomic characteristics. Demographic variables included gender (trans man, trans woman, *travesti*, non-binary), race/skin color (Black, mixed-race, Asian, White, Indigenous), Age ( at last birthday), and marital status (single, married, widowed, separated, with a partner). The socioeconomic variables analyzed were education level (elementary education, high school, higher education), paid work (yes, no), occupation (unemployed, public servant, employee, self-employed, employer), total family income (<1 minimum wage, >1-2 minimum wages, >2-5 minimum wages, ≥5 minimum wages), social benefits (yes, no), whether they had ever been homeless (yes, no), and whether they had ever been in street situations (yes, no).

Developed in 1995,^11^ the SF-12 is a shortened version of the SF-36, consisting of 12 items that assess, across 8 different domains, the individual’s perception of aspects of their health in the last 4 weeks.^21^ Each item offers a set of possible dichotomous responses (yes, no) or is distributed on a Likert- type scale, with 3 to 6 options, depending on the question, which reflected different degrees of intensity or frequency, ranging, for example, from “excellent” to “poor” or “all the time” to “never”. This structure allowed for capturing variations in the interviewees’ perception across the domains that make up the instrument: functional capacity, physical aspects, pain, general health status, vitality, emotional aspects, social aspects and mental health. Through the application of the instrument’s algorithm, 2 scores were measured: the physical component and the mental component. The score ranges from 0 to 100, with higher scores associated with better quality of life.^11^

A pilot study was conducted with 7 trans people, not included in the main study, to train the examiners and verify the feasibility of applying the instrument to the study population, as well as to verify the clarity and comprehensibility of the questionnaire items.

### Study size

Based on the service administrative records and the flow of active people who were receiving care, a sample size of 70 participants was estimated, which represents a power of 80% to estimate effects of 0.2 in a regression model with five covariates at a significance level of 5%.

### Statistical methods

The data were tabulated in Excel spreadsheets and imported into the software Stata SE, version 15. After calculating the SF-12 scores using the instrument’s syntax, descriptive analyses were performed. Numerical variables were described using the mean and standard deviation, and categorical variables were described by means of absolute and relative frequencies. A radar chart was used to summarize the values of the domains of the quality-of-life instrument. The scores of the SF-12 components were compared by estimating the mean and 95% confidence intervals (95%CI) between two categories: trans men and trans women and *travestis*, given that they embody and express femininity.

A non-parametric Kernel regression analysis was performed to assess the association between sociodemographic variables and component scores, estimating mean differences (MD) and 95%CI, calculated using the bootstrap method. Variables with p-value<0.20 in the bivariate regression analyses were included in the multivariable model. In the final models, variables with p-value<0.10 were retained and the significance level was set at 0.05.

## RESULTS

A total of 71 trans people were evaluated, with a mean age of 30.1±8.1 years. Half of the sample (n=36) identified as trans women, while 29 participants identified as trans men. The majority of participants self-reported as Black or mixed-race (n=48), single (n=53), with high school education (n=44) and an average household income between >1-2 minimum wages. Twenty-five individuals reported having experienced homelessness and 13 had lived in street situations. Table 1 presents the sociodemographic characteristics of the study participants.

**Table 1:** Sociodemographic characteristics of trans people using a specialized health service, Manaus, Amazonas state, Brazil, 2023 (n=71)

Variable	n
**Gender**	
Trans man	29
Trans woman	36
*Travesti*	2
Non-binary person	4
**Race/skin color**	
Black	6
Mixed-race	42
Asian	1
White	19
Indigenous	3
**Marital status**	
Single	53
Married	7
Widowed	1
Separated	1
With a partner	9
**Education level**	
Elementary education	12
High school	44
Higher education	15
**Paid employment**	
No	35
Yes	36
**Occupation**	
Unemployed	34
Public servant	8
Employee	11
Self-employed	17
Employer	1
**Total household income (minimum wages)**	
<1	24
>1-2	27
>2-5	16
≥5	4
**Social benefit**	
Yes	41
No	30
**Has ever been homeless**	
Yes	25
No	46
**Has ever** **lived in street situations**	
Yes	13
No	58

The first question of the SF-12 refers to the self-perception of the general health of the study population and was not used to calculate the scores. Half of the participants considered their general health to be good (n=36), while 13 perceived their health as excellent or very good and 22 rated it as poor or very poor. 

**Figure 1:**
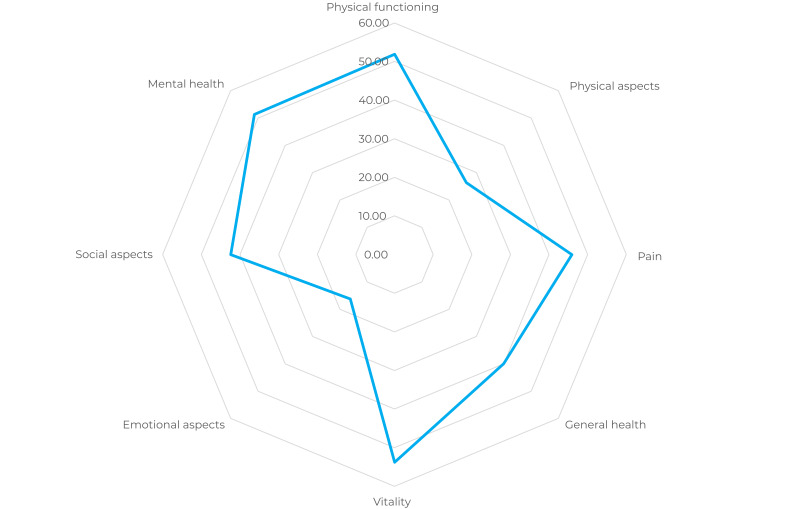
Mean scores of the domains of the health-related quality of life instrument in trans people using a specialized health service, Manaus, Amazonas state, Brazil 2023 (n=71)

Table 2 presents the scores for the physical and mental components, as well as for each of the 8 domains. The average score observed was 44.6 for the physical and 38.4 for the mental component. The emotional aspect showed the lowest score (16.0), making it the most affected domain, followed by the physical aspect (26.0). The 3 domains with the highest scores were: vitality (52.1), mental health (52.1) and physical functioning (52.0). The scores for the domains of the SF-12 instrument are summarized in Figure 1.

**Table 2:** Mean and standard deviation (SD), minimum and maximum values of health-related quality of life in trans people using a specialized health service, Manaus, Amazonas state, Brazil, 2023 (n=71)

Components/domains	Mean ± SD	Minimum	Maximum
**Physical component**	44.6±7.8	16.8	58.3
Physical functioning	52.0±9.2	22.1	56.5
Physical appearance	26.0±4.0	20.3	29.5
Pain	46.1±11.9	16.7	57.4
General health	38.9±9.7	18.9	62.0
**Mental component**	38.4±11.1	13.1	60.7
Vitality	52.1±14.4	27.6	77.9
Emotional aspect	16.0±1.1	11.3	22.5
Social aspect	44.8±15.3	16.2	66.7
Mental health	52.1±13.6	21.9	76.7

Based on the estimated confidence intervals, a lower mean score for the mental component compared to the physical component was observed among trans people. No significant difference was found between genders in the SF-12 component score (Table 3).

**Table 3:** Mean scores (95% confidence interval) of the physical and mental components in trans people using a specialized health service, according to gender identity, Manaus, Amazonas state, Brazil, 2023

Components	Trans woman or *travesti* (n=31) MS (95%CI)	Trans man (n=36) MS (95%CI)	Trans people (n=71) MS (95%CI)
Physical	44.2 (41.1;47.4)	44.7 ( 42.2;47.2)	44.6 (42.7;46.4)
Mental	39.9 (35.6;44.2)	37.5 (33.9;41.1)	38.4 (35.8;41.0)

Trans people who reported having paid employment showed higher scores on the mental component of the SF-12 (mean difference 4.4; 95%CI 2.3;6.9). Having lived in street situations was associated with lower physical component scores on the SF-12 (mean difference -3.3; 95%CI -7.7;-2.3) (Table 4).

**Table 4:** Crude and adjusted mean difference (MD) and 95% confidence interval (95%CI) of the mental and physical components of the quality of life in trans people using a specialized health service, Manaus, Amazonas state, Brazil, 2023 (n=71)

Variable	Mental component			Physical component		
	Crude MD (95%CI)	Adjusted MD (95%CI)	p-value	Crude MD (95%CI)	Adjusted MD (95%CI)	p-value
**Gender**						
Trans man	-0.2 (-0.6;0.2)			0.0 (-0.1;0.1)		
Trans woman or *travesti*	0.00			0.00		
**Age (years)**	0.3 (-0.1;0.7)			0.0 (-0.3;0.3)		
**Income (minimum wages)**						
≤1	-1.5 (-3.4;0.3)			0.0 (-0.1;0.1)		
>1	0.00			0.00		
**Education level**						
Elementary education	0.1 (-0.2;0.4)			0.0 (-0.1;0.0)		
High school/higher education	0.00			0.00		
**Paid employment**						
Yes	4.7 (0.6;8.2)	4.4 (2.3;6.9)	0.026	-1.1 (-2.3;0.4)	-0.5 (-1.2;0.0)	0.105
No	0.00	0.00		0.00	0.00	
**Partner/spouse**						
Yes	2.2 (-0.2;4.8)	0.6 (-0.1;1.0)	0.118	0.0 (0.0;0.1)		
No	0.00	0.00		0.00		
**Has ever been homeless**						
Yes	-1.1 (-2.4;0.3)			0.0 (-0.1;0.0)		
No	0.00			0.00		
**Has ever lived in street situations**						
Yes	0.4 (-0.5;0.9)			-3.6 (-8.3;0.5)	-3.3 (-7.7;-2.3)	0.019
No	0.00			0.00	0.00	

## DISCUSSION

In the assessment of the quality of life of trans people, the mental component was worse than the physical component, with “emotional aspect” being the most affected domain. No difference in quality of life was found when comparing trans men with trans women and *travestis*. Trans people who reported having paid employment showed a better mental component of quality of life, while having lived in street situations was associated with a worse physical component. One-third of trans people perceived their health as poor or very poor.

The scores for the mental and physical components of quality of life of trans people assessed in this study were lower than those of other trans populations in India,^5^ the United States,^22^ and Italy.^6^ Higher quality of life scores have also been observed in studies involving trans women who have undergone gender-affirming surgery^13^ and trans men.^15^ Results of a systematic review showed that trans people have impaired quality of life and report worse mental quality of life compared to the general population.^4^

The scores in this study were worse than those of a representative sample of individuals aged 15 years or older from 15 capital cities across the five regions of Brazil, including Manaus, which reported a mean score of 49.3 (95%CI 49.1;49.6) for the physical component of the SF-12 and 52.7 (95%CI 52.4;52.9) for the mental component.^12^ In the Brazilian population survey, the physical component of quality of life was more affected than the mental component, unlike what was found for trans people in this study. These people showed worse mental component than physical component, which aligns with findings from other studies that also assessed trans people in international contexts.^5-6,15,24-25^

The transsexualizing process is marked by biological and physiological factors, sexual characteristics, and psychological impairments, which includes anxiety, depression, suicidal ideation,^7^ as well as social issues, such as lack of social support, rejection, discrimination and transphobia.^1,4^-7 Trans people face barriers to accessing healthcare services, which are often structured around binary and heteronormative systems that prevent this population from seeking care. Even when they manage to access healthcare, they face assistance from professionals who fail to recognize their needs.^24^ All these exposures can explain the negative impact on the quality of life of these people, in addition to justifying the greatest negative impact in the “emotional aspect” domain, which was also one of the most affected in other trans populations.^15^ It is worth highlighting that the study did not distinguish the trans people investigated in relation to their stage in the transsexualizing process. There is evidence that quality of life improves after gender affirming treatment.^4,14^

No differences in the physical and mental components of quality of life were observed between trans men and trans women (the latter assessed together with *travestis*). A study conducted between 2016 and 2017 in a healthcare service in Porto Alegre, capital city of Rio Grande do Sul state, also found no differences between genders,^18^ while demonstrating that factors related to the body and the transsexualizing process associated with quality of life were different for trans men and women. This suggests a similarity in the perception of physical health between the two groups, indicating that the factors influencing physical health experiences may be shared, challenging existing stereotypes about the specific experiences of transgender men and women in this dimension. Globally, trans men and trans women face similar challenges in terms of mental health.

Trans people with paid employment showed better mental quality of life, while those who lived in street situations had worse physical quality of life. Socioeconomic factors such as education level,^14,17-18^ income, and occupation were found to be associated with the quality of life of transgender individuals, similar to what is observed in the general population.^19,20^ Social stratification is associated with differences in exposure and vulnerability to health-compromising conditions. This stratification is also associated with different ways of coping with and the consequences of health problems, constituting the fundamental mechanism through which socioeconomic position generates health inequalities.^25^

Although the associations identified reflect vulnerabilities that may be common to the general population, for the trans population, these vulnerabilities are exacerbated by specific intersectional factors, such as gender discrimination, social exclusion and barriers to accessing health services. *Travestis* and trans women living in street situations were able to access services from the Brazilian National Health System and the Brazilian National Social Assistance System in Belo Horizonte, capital city of Minas Gerais state, in a study conducted between October 2017 and February 2018, but faced challenges due to inadequacy of services to meet their needs, which highlights gaps in social protection and health care provision.^26^

One-third of trans people perceived their health as poor or very poor. Like quality of life, self-perceived health is a subjective indicator that reflects an integration of functional health status with emotional distress and social factors. Quality of life is a predictor of the use of general and mental healthcare services and life expectancy. General health perception is certainly related to biological and physiological factors, but understanding that other factors affect health perceptions may explain variations in each clinical state or between individuals.^10^ Five percent of the Brazilian population reported poor or very poor self-perceived health in 2019.^27^ The proportion of trans people with poor self-perceived health found in this study reiterates and contributes to justifying the negative assessment of quality of life.

Some limitations of the study should be noted. The sample was non-probabilistic and involved only users of a reference center for the transsexualizing process, which represents selection bias by excluding people who were unable to access the healthcare service. Recruiting participants from healthcare services is common in studies involving the trans population, although the possibility of systematic bias arising from this is acknowledged.^4^

The low number of participants may not have been sufficient to identify differences between evaluated categories that may exist. The trans population is highly vulnerable, with challenges in recruiting and retaining participants resulting in variability in planning and sample size.^4, 14^ There was no distinction regarding the therapeutic stage of the transsexualizing process in which individuals were. This deserves investigation concerning quality of life, particularly in conjunction with a qualitative investigation, considering that there is evidence that quality of life can improve after intervention^s. 28^

The study suggests that including measures of subjective perceptions in the context of the transsexualizing process would help assess the relationship between gender identity and the physical and psychological changes that accompany the transition. One of the main goals of treatment, in addition to body changes, is to improve the quality of life and social integration of trans people.^29^ This cannot be assessed solely by normative clinical measures, which neglect the needs perceived by individuals, as well as their potential psychosocial implications. These self-reported subjective measures describe or characterize the individual’s perception of their own health, considering the physical, psychological and social aspects involved, or what the individual has experienced as a result of medical care. These measures complement traditional biological or physiological health state metrics.

This study found that the health-related quality of life of trans people using a specialized service in Manaus was worse in the mental component, with the emotional aspect being the most affected. Socioeconomic factors, such as paid employment and having lived in street situations, were associated with better mental and worse physical quality of life outcomes, respectively. These findings reinforce the vulnerability of the trans population and the importance of considering subjective and psychosocial factors in healthcare provision.
