# Supplementary material for: Health-related quality of life of trans people using a specialized health service in Manaus, Amazonas state, Brazil, 2023: a cross-sectional study
Source: Epidemiol Serv Saude. 2025 Jan 10;33(spe1):e2024361. doi: 10.1590/S2237-96222024v33e2024361.especial.en (PMC11734657; doi:10.1590/S2237-96222024v33e2024361.especial.en)
Supplement: Translation

# Translation: Qualidade de vida relacionada à saúde de pessoas trans usuárias de serviço de saúde especializado em Manaus, 2023: um estudo transversal

**DOI:** 10.1590/S2237-96222024v33e2024361.especial.pt

**Authors:** Erivan Clementino Gualberto, Açucena Amâncio Dall’Alba, André Luiz Machado das Neves, David Câmara Gurgel do Amaral, Aline Barreto Soares, Fernando José Herkrath, Ana Paula Corrêa de Queiroz Herkrath

**Affiliations:** 1 Universidade Federal do Amazonas, Faculdade de Odontologia, Manaus, AM, Brasil; 2 Universidade do Estado do Amazonas, Escola Superior de Ciências da Saúde, Manaus, AM, Brasil; 3 Fundação Oswaldo Cruz, Instituto Leônidas e Maria Deane, Manaus, AM, Brasil

**Keywords:** Pessoas Transgênero, Qualidade de Vida Relacionada à Saúde, Serviços de Saúde para Pessoas Transgênero, Estudos Transversais

## Abstract

**Objetivo:**

Avaliar a qualidade de vida relacionada à saúde e fatores associados em pessoas trans.

**Métodos:**

Estudo transversal em 2023. Empregou-se o instrumento *12-Item Short-Form Health Survey* em mulheres trans, homens trans, travestis e não binários, usuários de um serviço de saúde em Manaus.

**Resultados:**

Incluídos 71 participantes: 36 mulheres trans; 29, homens trans; 2, travestis; e 4, não binários, média de idade 30,1 anos. O componente físico apresentou escore 44,6 ±7,8, e o componente mental, 38,4±11,1. O aspecto emocional, escore 16,0±1,1, foi o domínio mais afetado. Associaram-se trabalho remunerado com melhor qualidade de vida mental e ter vivido em situação de rua com pior qualidade de vida física, diferenças médias de 4,4 (IC_95_% 2,3;6,9) e -3,3 (IC_95_% -7,7;-2,3) respectivamente.

**Conclusão:**

Em pessoas trans, o componente mental foi mais afetado que o físico, com maior prejuízo no aspecto emocional. Características socioeconômicas associaram-se à qualidade de vida.

## Contribuições do estudo

**Principais resultados:**

O componente mental da qualidade de vida das pessoas trans avaliadas foi pior do que o físico, com maior prejuízo no aspecto emocional. As pessoas trans com trabalho remunerado relataram melhor qualidade de vida, enquanto ter vivido em situação de rua foi associado à pior.

**Implicações para os serviços:**

A qualidade de vida favorece a compreensão do estado de saúde autopercebido e deve ser considerada como desfecho de saúde. Pode-se explicitar necessidades não contempladas pelos serviços e fomentar a integralidade, a inclusão e o acolhimento na atenção à saúde.

**Perspectivas:**

Os serviços de saúde e a avaliação de intervenções terapêuticas em pessoas trans devem incorporar desfechos de saúde autorreferidos. Estes possibilitam avaliar a qualidade do cuidado e subsidiar ações de saúde sensíveis às peculiaridades dessa população.

## INTRODUÇÃO

Conceitualmente, o termo “trans” abrange todas as pessoas que não se identificam com as expressões sociais tradicionais dicotômicas de gêner^o^.^1^ Diante da pluralidade das experiências das pessoas travestis, transexuais e transgêneros, “trans” é utilizado como termo guarda-chuva, que integra diferentes identidades e funcionalidades, compondo um grupo diverso de pessoas que neste estudo serão denominados de “pessoas trans^”^.^1^

A população mundial que se autoidentifica como pessoa trans é de 4,6 em 100 mil pessoas, sendo maior para mulheres trans (6,8 em 100 mil) do que para homens trans (2,6 em 100 mil^)^.^2^ No Brasil a diversidade de gênero representa cerca de 2% da população adulta do país. Estima-se que 3 milhões de brasileiros identificam-se como pessoas trans. São pessoas, em média, mais jovens (32,9±13,5) quando comparados aos cisgêneros (42,2±15,9). A média de idade dessas pessoas pode estar associada à menor expectativa de vida dessa populaçã^o^.^3^

O desrespeito ao nome social e a incapacidade de gerir questões específicas da população tran^s^,^1^ bem como a dificuldade de inserção no mercado de trabalho, renda, conflitos sociais e familiare^s,4^-6 permeiam a realidade no acesso a bens e serviços por pessoas trans. Grande parte dos problemas de saúde dessa população está direta e indiretamente relacionada à exposição ao preconceito, à discriminação e à violênci^a^.^7^ Depressão, ansiedade, estresse, uso de substâncias nocivas à saúde, automutilação e tentativas de suicíd^i^o^7^ acometem frequentemente esses indivíduos. Atitudes discriminatórias afetam a relação entre médico e paciente e prejudica ações de educação e prevenção em saúd^e^.^1^ Tais exposições afetam a qualidade de vida dessa população. 

A Organização Mundial da Saúde define a qualidade de vida como a autopercepção sobre sua condição de vida, no seu próprio contexto de cultura e sistema de valores, considerando seus objetivos de vida, as expectativas e as preocupaçõe^s^.^8^ Mantendo-se a subjetividade e a multidimensionalidade da qualidade de vida, surgiu o conceito de qualidade de vida relacionada à saúde, que é definida pelos aspectos da qualidade de vida que se relacionam direta ou indiretamente com a saúd^e^.^9^ A compreensão da qualidade de vida é focada na avaliação subjetiva da pess^o^a^9^ e pode ser avaliada através de instrumentos em forma de questionários que investigam o impacto das condições de saúde com relação aos aspectos físicos, psicológicos, emocionais e sociais de cada pesso^a.10-^ 12 Ao longo deste artigo, o termo “qualidade de vida” foi empregado para se referir ao desfecho “qualidade de vida relacionada a saúde”.

A qualidade de vida de pessoas trans mostrou-se pior que a de pessoas cisgênero em pesquisas prévia^s.4,13-^ 15 Pessoas trans, quando comparadas à população cisgênero, experienciam diversas situações de discriminação que interferem negativamente no seu cotidian^o.^ 16 O estresse psicossocial tem o potencial de afetar os domínios físico, psicológico e de bem-estar, que corroboram o comprometimento da qualidade de vid^a.7,^ 16 Fatores como tratamento com terapia hormonal de reafirmação de gêner^o,17,^ 18 relação amorosa estáve^l,^ 18 trabalhar ou estud^ar14,^ 18 estão associados a melhores escores de qualidade de vida. Fatores demográficos e socioeconômicos também têm sido associados à qualidade de vida de pessoas tran^s,14,17-^ 18 assim como acontece com a população em gera^l.19-^ 20

Estudos que investiguem a qualidade de vida na população trans ainda são escassos. Este estudo objetivou avaliar a qualidade de vida relacionada à saúde e os fatores associados em pessoa trans em Manaus, Amazonas.

## MÉTODOS

### Desenho do estudo

Trata-se de um estudo transversal aprovado pelo Comitê de Ética em Pesquisa da Universidade Federal do Amazonas por meio do parecer 6.315.703, com certificado de apresentação para apreciação ética nº 65732222.0.0000.5020. Todos os participantes assinaram o Termo de Consentimento Livre e Esclarecido, conforme as diretrizes éticas estabelecidas pela Resolução nº 466/12 do Conselho Nacional de Saúde.

### Contexto

O estudo foi realizado no período de abril a dezembro de 2023 no Ambulatório de Diversidade Sexual e Gênero, localizado na Policlínica Codajás, em Manaus, Amazonas, serviço de referência da Secretaria de Estado de Saúde que oferta assistência realizada por uma equipe multidisciplinar durante o processo transexualizador.

### Participantes

A amostra de conveniência foi composta por pessoas acima de 18 anos que se autoidentificaram como homens trans, mulheres trans, travestis e não binários, usuários do Ambulatório de Diversidade Sexual e Gênero. O critério de exclusão foi estar sob efeito de substância psicoativa ou álcool no momento da entrevista de forma a impossibilitar a aplicação do questionário. A participação no processo transexualizador, decisão de participar ou momento terapêutico do processo não foram considerados para elegibilidade no estudo. 

As pessoas foram convidadas a participar na recepção, durante a espera pelo atendimento no serviço de saúde. Esse primeiro contato foi auxiliado pelos profissionais do ambulatório. Aquelas pessoas que concordaram em participar e atenderam aos critérios de elegibilidade foram direcionadas a uma sala reservada para obtenção do consentimento livre esclarecido e coleta de dados.

### Variáveis, fonte de dados e mensuração

Os dados foram coletados utilizando-se questionários aplicados por meio de entrevistas, conduzidas por 3 entrevistadores treinados. O desfecho do estudo foi a qualidade de vida relacionada à saúde, mensurado pelo instrumento Short-Form Health Survey (SF-12) na sua versão traduzida e validada para o português do Brasi^l.^ 21 As exposições avaliadas foram características demográficas e socioeconômicas. As variáveis demográficas incluíram gênero (homem trans, mulher trans, travesti, não binário), raça/cor da pele (preta, parda, amarela, branca, indígena), idade (em anos) e estado civil (solteiro(a), casado(a), viúvo(a), separado(a), com companheiro(a)). As variáveis socioeconômicas analisadas foram escolaridade (fundamental, médio, superior), trabalho remunerado (sim, não), ocupação (desempregado(a), servidor(a) público(a), empregado, autônomo, empregador), renda familiar total (<1 salário mínimo, >1 -2 salários mínimos, >2 –5 salários mínimos, ≥5 salários mínimos), benefício social (sim, não), se já ficou sem ter onde morar (sim, não) e se já esteve em situação de rua (sim, não).

Elaborado em 199^5,^ 11 o SF-12 é uma versão reduzida do SF-36, composta por 12 itens que avaliam, em 8 diferentes domínios, a percepção do indivíduo em relação aos aspectos de sua saúde nas 4 últimas semana^s.^ 21 Cada item apresentou um conjunto de possíveis respostas dicotômicas (sim, não) ou distribuídas em uma escala gradativa tipo Likert, abrangendo de 3 a 6 opções de escolha, dependendo da questão, as quais refletiram diferentes graus de intensidade ou frequência, variando, por exemplo, de “excelente” a “ruim” ou “todo o tempo” a “nenhuma parte do tempo”. Essa estrutura permitiu captar variações na percepção dos entrevistados em relação aos domínios que compõem o instrumento: capacidade funcional, aspectos físicos, dor, estado geral de saúde, vitalidade, aspectos emocionais, aspectos sociais e saúde mental. Por meio da aplicação de um algoritmo próprio do instrumento, 2 escores foram mensurados: o componente físico e o componente mental. A pontuação varia de 0 a 100, sendo os maiores escores associados à melhor qualidade de vid^a.^ 11

Um estudo piloto foi realizado com 7 pessoas trans, não incluídas no estudo principal, para treinamento dos examinadores e verificação da viabilidade da aplicação do instrumento na população do estudo, bem como averiguação da clareza e compreensibilidade dos itens do questionário.

### Tamanho do estudo

Com base nos registros administrativos do serviço e no fluxo de pacientes ativos, estimou-se tamanho amostral de 70 participantes, o que representa um poder de 80% em estimar efeitos de 0,2 em um modelo de regressão com cinco covariáveis no nível de significância de 5%.

### Métodos estatísticos

Os dados foram tabulados em planilha de Excel e importados no *software* Stata SE, versão 15. Após o cálculo dos escores do SF-12, por meio da sintaxe do instrumento, foram realizadas as análises descritivas. As variáveis numéricas foram descritas pela média e desvio padrão, e as variáveis categóricas, pelas frequências absoluta e relativa. Um gráfico de radar foi utilizado para sumarizar os valores dos domínios do instrumento de qualidade de vida. Os escores dos componentes do SF-12 foram comparados por meio da estimativa da média e intervalos de confiança de 95% (IC_95_%) entre duas categorias: homens trans e mulheres trans e travestis, uma vez que elas vivem e carregam em si as expressões do feminino. 

Realizou-se análise de regressão não Paramétrica Kernel para avaliar a associação entre variáveis sociodemográficas e os escores dos componentes, estimando as diferenças médias (DM) e IC_95_%, calculados por método de reamostragem. As variáveis com p-valor<0,20 nas análises de regressão bivariadas foram incluídas no modelo múltiplo. Nos modelos finais, mantiveram-se as variáveis com p-valor<0,10 e estabeleceu-se o nível de significância em 0,05.

## RESULTADOS

Foram avaliadas 71 pessoas trans, com média de idade de 30,1±8,1 anos. Metade da amostra (n=36) se autoidentificou como mulher trans, enquanto 29 participantes se identificaram como homens trans. A maioria se autodeclarou da raça preta ou parda (n=48), de estado civil solteiro(a) (n=53), com ensino médio (n=44) e renda média familiar entre >1-2 salários mínimos. Vinte e cinco pessoas relataram já ter ficado sem ter onde morar e 13 em situação de rua. A Tabela 1 apresenta a caracterização sociodemográfica dos participantes do estudo.

**Tabela 1:** Caracterização sociodemográfica de pessoas trans usuárias de serviço de saúde especializado, Manaus, Amazonas, 2023 (n=71)

Variável	n
**Gênero**	
Homem trans	29
Mulher trans	36
Travesti	2
Não binário	4
**Raça/cor da pele**	
Preta	6
Parda	42
Amarela	1
Branca	19
Indígena	3
**Estado civil**	
Solteiro	53
Casado	7
Viúvo	1
Separado	1
Com companheiro	9
**Escolaridade**	
Fundamental	12
Médio	44
Superior	15
**Trabalho remunerado**	
Não	35
Sim	36
**Ocupação**	
Desempregado	34
Servidor público	8
Empregado	11
Autônomo	17
Empregador	1
**Renda familiar total (salários mínimos)**	
<1	24
>1-2	27
>2-5	16
≥5	4
**Benefício social**	
Sim	41
Não	30
**Já ficou sem ter onde morar**	
Sim	25
Não	46
**Já esteve em situação de rua**	
Sim	13
Não	58

A primeira questão do SF-12 refere-se à autopercepção da saúde geral da população de estudo, não sendo utilizada para o cálculo dos escores. Metade dos participantes considerou sua saúde geral como boa (n=36), enquanto 13 perceberam sua saúde como excelente ou muito boa e 22 como ruim ou muito ruim. 

A Tabela 2 apresenta os escores dos componentes físico e mental, bem como de cada um dos 8 domínios. O escore médio observado foi 44,6 para o físico e 38,4 para o mental. O aspecto emocional apresentou o menor escore (16,0), sendo o domínio mais afetado, seguido do aspecto físico (26,0). Os 3 domínios com as maiores pontuações foram: vitalidade (52,1), saúde mental (52,1) e capacidade física (52,0). A pontuação dos domínios do instrumento SF-12 foi sumarizada na Figura 1.

**Figura 1:** Escores médios dos domínios do instrumento de qualidade de vida relacionada à saúde em pessoas trans usuárias de serviço de saúde especializado, Manaus, Amazonas, 2023 (n=71)

**Tabela 2:** Média e desvio padrão (DP), valores mínimos e máximos da qualidade de vida relacionada à saúde em pessoas trans usuárias de serviço de saúde especializado, Manaus, Amazonas, 2023 (n=71)

Componentes/domínios	Média ± DP	Mínimo	Máximo
**Componente físico**	44,6±7,8	16,8	58,3
Capacidade física	52,0±9,2	22,1	56,5
Aspecto físico	26,0±4,0	20,3	29,5
Dor	46,1±11,9	16,7	57,4
Saúde geral	38,9±9,7	18,9	62,0
**Componente mental**	38,4±11,1	13,1	60,7
Vitalidade	52,1±14,4	27,6	77,9
Aspecto emocional	16,0±1,1	11,3	22,5
Aspecto social	44,8±15,3	16,2	66,7
Saúde mental	52,1±13,6	21,9	76,7

Por meio dos intervalos de confiança estimados, observou-se menor valor no escore médio do componente mental em relação ao componente físico em pessoas trans. Não foi observada diferença significativa nos escores dos componentes do SF-12 entre os gêneros (Tabela 3).

**Tabela 3:** Tabela 3 **– Média do escore (intervalo de confiança de 95%) dos componentes físico e mental do de pessoas trans usuárias de serviço de saúde especializado, de acordo com a identidade de gênero, Manaus, Amazonas, 2023**

Componentes	Mulher trans ou travesti (n=31) ME (IC_95_ **%**)	Homem trans (n=36) ME (IC_95_ **%**)	Pessoas trans (n=71) ME (IC_95_ **%**)
Físico	44,2 (41,1;47,4)	44,7 (42,2;47,2)	44,6 (42,7;46,4)
Mental	39,9 (35,6;44,2)	37,5 (33,9;41,1)	38,4 (35,8;41,0)

As pessoas trans que referiram ter trabalho remunerado apresentaram melhores escores no componente mental do SF-12 (diferença média 4,4; IC_95_% 2,3;6,9). Ter vivido em situação de rua foi associado com pior escore do componente físico do SF-12 (diferença média -3,3; IC_95_% -7,7;-2,3) (Tabela 4).

**Tabela 4:** Diferença média (DM) e intervalo de confiança de 95% (IC_95_%) bruta e ajustada dos componentes mental e físico da qualidade de vida de pessoas trans usuárias de serviço de saúde especializado, Manaus, Amazonas, 2023 (n=71)

Variável	Componente mental			Componente físico		
	DM bruta (IC _95_ % )	DM ajustada (IC _95_ % )	p-valor	DM bruta (IC _95_ % )	DM ajustada (IC _95_ % )	p-valor
**Gênero**						
Homem trans	-0,2 (-0,6;0,2)			0,0 (-0,1;0,1)		
Mulher trans ou travesti	0,00			0,00		
Idade (anos)	0,3 (-0,1;0,7)			0,0 (-0,3;0,3)		
**Renda (salários mínimos)**						
≤1	-1,5 (-3,4;0,3)			0,0 (-0,1;0,1)		
>1	0,00			0,00		
**Escolaridade**						
Fundamental	0,1 (-0,2;0,4)			0,0 (-0,1;0,0)		
Médio/superior	0,00			0,00		
Trabalho remunerado						
Sim	4,7 (0,6;8,2)	4,4 (2,3;6,9)	0,026	-1,1 (-2,3;0,4)	-0,5 (-1,2;0,0)	0,105
Não	0,00	0,00		0,00	0,00	
**Companheiro/cônjuge**						
Sim	2,2 (-0,2;4,8)	0,6 (-0,1;1,0)	0,118	0,0 (0,0;0,1)		
Não	0,00	0,00		0,00		
**Já ficou sem ter onde morar**						
Sim	-1,1 (-2,4;0,3)			0,0 (-0,1;0,0)		
Não	0,00			0,00		
**Já esteve em situação de rua**						
Sim	0,4 (-0,5;0,9)			-3,6 (-8,3;0,5)	-3,3 (-7,7;-2,3)	0,019
Não	0,00			0,00	0,00	

## DISCUSSÃO

Na avaliação da qualidade de vida de pessoas trans, o componente mental apresentou-se pior que o componente físico, tendo sido “aspecto emocional” o domínio mais afetado. Não houve diferença na qualidade de vida ao se comparar homens trans com mulheres trans e travestis. As pessoas trans que referiram ter trabalho remunerado apresentaram melhor componente mental da qualidade de vida, enquanto ter vivido em situação de rua foi associado com pior componente físico. Um terço das pessoas trans percebeu sua saúde como ruim ou muito ruim.

Os escores dos componentes mental e físico da qualidade de vida das pessoas trans avaliadas neste estudo foram menores que os de outras populações transgênero na Índi^a^,^5^ nos Estados Unid^os^ 22 e na Itáli^a^.^6^ Também observaram-se maiores escores de qualidade de vida em pesquisas com mulheres trans submetidas à cirurgia de redesignação sexu^al^ 13 e homens tran^s.^ 15 Os resultados de uma revisão sistemática mostraram que pessoas transgênero apresentam prejuízo na qualidade de vida e que relatam pior qualidade de vida mental em comparação à população em gera^l^.^4^

Os escores deste estudo foram piores que os de uma amostra representativa para 15 capitais das 5 regiões brasileiras, incluindo Manaus, de indivíduos com 15 anos ou mais, que observou média de 49,3 (IC_95_% 49,1;49,6) no componente físico do SF-12 e 52,7 (IC_95_% 52,4;52,9) no componente menta^l.^ 12 No levantamento da população brasileira, o componente físico da qualidade de vida mostrou-se mais afetado que o componente mental, diferentemente do encontrado para as pessoas trans deste estudo. Estas tiveram o componente mental pior que o físico, o que está em concordância com outros estudos que também avaliaram pessoas trans em contextos internacionai^s.5-6,15,24-^ 25

O processo transexualizador é marcado por fatores biológicos e fisiológicos, as características sexuais, e por prejuízos psicológicos, que incluem ansiedade, depressão, ideação suicid^a^,^7^ e sociais, como falta de apoio social, rejeição, discriminação e transfobi^a.1,4^-7As pessoas trans sofrem com as barreiras de acesso ao serviço de saúde, características do sistema binário e heteronormativo, que impedem que essa população procure os serviços de saúde. Quando esta consegue acessar e utilizar o serviço de saúde, padece com a assistência de profissionais que invisibilizam suas necessidade^s.^ 24 Todas essas exposições podem explicar o impacto negativo na qualidade de vida dessas pessoas, além de poderem justificar o maior impacto negativo no domínio “aspecto emocional”, que também foi ou o mais ou um dos mais afetados em outras populações tran^s.^ 15 É importante ressaltar que não foi feita distinção das pessoas trans investigadas em relação ao momento do processo transexualizador em que se encontravam. Há evidência que a qualidade de vida melhora após o tratamento de redesignação sexua^l.4,^ 14

Não foram observadas diferenças nos componentes físico e mental da qualidade de vida entre homens trans e mulheres trans (avaliadas em conjunto com as travestis). Pesquisa realizada entre 2016 e 2017 em um serviço de saúde de Porto Alegre (RS) também não encontrou diferenças entre os gênero^s,^ 18 ao mesmo tempo que demonstrou que fatores relacionados ao corpo e ao processo transexualizador associados à qualidade de vida eram diferentes para homens e mulheres trans. Isso sugere uma similaridade na percepção da saúde física entre os dois grupos, o que pode apontar que os fatores na experiência de saúde física são compartilhados, desafiando estereótipos preexistentes sobre as vivências específicas de homens e mulheres trans nessa dimensão. Globalmente, homens trans e mulheres trans enfrentam desafios semelhantes em termos de saúde mental.

As pessoas trans que tinham trabalho remunerado apresentaram melhor qualidade de vida, no seu componente mental, e aquelas que viveram em situação de rua tiveram pior componente físico. A associação de fatores socioeconômicos, como escolaridade, renda e ocupaçã^o,14,17-^ 18 mostraram-se associados à qualidade de vida de pessoas trans, da mesma forma que na população em gera^l.19,^ 20 A estratificação social está associada a diferenciais de exposição e vulnerabilidade a condições comprometedoras da saúde. Essa estratificação se associa também a diferentes formas de enfrentamento e consequências dos problemas de saúde, constituindo-se esse o mecanismo fundamental pelo qual a posição socioeconômica gera desigualdades na saúd^e.^ 25

Embora as associações identificadas reflitam vulnerabilidades que podem ser comuns a população em geral, para a população trans, essas vulnerabilidades são agravadas por fatores interseccionais específicos, como a discriminação de gênero, a exclusão social e as barreiras de acesso a serviços de saúde. Travestis e mulheres trans em situação de rua conseguiram acessar serviços do Sistema Único de Saúde e do Sistema Único de Assistência Social no município de Belo Horizonte (MG) em pesquisa realizada entre outubro de 2017 a fevereiro de 2018, mas enfrentaram desafios de inadequação às suas necessidades, o que evidencia lacunas na proteção social e na produção do cuidado em saúd^e.^ 26

Um terço das pessoas trans percebeu sua saúde como ruim ou muito ruim. Assim como a qualidade de vida, a autopercepção da saúde é um indicador subjetivo que reflete uma integração do estado funcional de saúde com o sofrimento emocional e os fatores sociais. A qualidade de vida é preditora do uso de serviços de saúde geral e mental e expectativa de vida. A percepção geral da saúde certamente está relacionada a fatores biológicos e fisiológicos, mas compreender que há outros fatores que afetam as percepções de saúde pode explicar as variações em cada estado clínico ou entre indivíduo^s.^ 10 Cinco por cento da população brasileira referiu autopercepção da saúde ruim ou muito ruim em 201^9.^ 27 A proporção de pessoas trans com autopercepção da saúde ruim encontrado no estudo reitera e contribui para justificar a avaliação negativa da qualidade de vida.

Algumas limitações do estudo devem ser apontadas. A amostra foi não probabilística e envolveu apenas usuários de um centro de referência no processo de transexualização, o que representa viés de seleção ao se excluírem pessoas que não conseguiram acesso ao serviço de saúde. O recrutamento dos participantes no serviço de saúde é usual em estudos com população trans, embora se reconheça a possibilidade de erro sistemático dele decorrent^e^.^4^

O pequeno número de participantes pode não ter tido o poder de identificar diferenças entre categorias avaliadas que podem, na realidade, existir. A população trans é altamente vulnerabilizada, com desafios em recrutar e reter os participantes resultando em variabilidade no planejamento e tamanho amostra^l.4,^ 14 Não houve distinção do momento terapêutico do processo transexualizador em que as pessoas estavam. Isso merece ser investigado em relação à qualidade de vida, inclusive em conjunto com uma investigação qualitativa, considerando que há evidências de que a qualidade de vida pode melhorar após as intervençõe^s.^ 28

O estudo sugere que a inclusão de medidas de percepções subjetivas no contexto do processo transexualizador ajudaria a avaliar a relação entre a identidade de gênero e as mudanças físicas e psicológicas que acompanham a transição. Um dos principais objetivos do tratamento, além das mudanças corporais, é o ganho na qualidade de vida e a integração social das pessoas tran^s.^ 29 Isso não pode ser avaliado apenas por medidas clínicas exclusivamente normativas, que negligenciam as necessidades percebidas pelas pessoas, bem como suas possíveis implicações psicossociais. Essas medidas subjetivas autorreportadas descrevem ou caracterizam a percepção da própria saúde, considerando os aspectos físicos, psicológicos e sociais que a permeiam, ou o que o paciente experienciou como resultado dos cuidados médicos. Tais medidas são complementares às medidas biológicas ou fisiológicas tradicionais do estado de saúde. 

O estudo identificou que a qualidade de vida relacionada à saúde de pessoas trans usuárias de um serviço especializado em Manaus apresentou-se pior no componente mental, sendo o aspecto emocional o mais afetado. Fatores socioeconômicos, como trabalho remunerado e ter vivido em situação de rua, estiveram associados, respectivamente, a melhor desfecho mental e pior desfecho físico da qualidade de vida. Esses achados reforçam a vulnerabilidade da população trans e a importância de considerar fatores subjetivos e psicossociais no cuidado à saúde.
